# Neferine and lianzixin extracts have protective effects on undifferentiated caffeine-damaged PC12 cells

**DOI:** 10.1186/s12906-020-2872-2

**Published:** 2020-03-06

**Authors:** Jingjing Chen, Mimi Tang, Manhua Liu, Yueping Jiang, Bin Liu, Shao Liu

**Affiliations:** 1 Department of Pharmacy, Xiangya Hospital, Central South University, Changsha, Hunan People’s Republic of China; 2 Institute for Rational and Safe Medication Practices, National Clinical Research Center for Geriatric Disorders, Xiangya Hospital, Central South University, Changsha, Hunan People’s Republic of China

**Keywords:** Lianzixin, Neferine, Alkaloid, PC12, Cell damage, Caffeine

## Abstract

**Background:**

The embryos of *Nelumbo nucifera* Gaertn seeds, lianzixin, are used in China as food and traditional herbal medicine. Principal therapeutic indications are insomnia, anxiety and pyrexia. Caffeine is a psychostimulant and excessive use predisposes to cell damage and neurotoxicity. We aimed to investigate the potential protect effect of Neferine and lianzixin extracts on undifferentiated caffeine-damaged phaeochromocytoma cells (PC12 cells).

**Methods:**

A cell damage model based on undifferentiated PC12 was established with caffeine. Effect of Lianzixin extracts (total alkaloids, alcohol extract and water extract) and neferine on caffeine-damaged PC12 cells was evaluated. Cell viability was assessed using the methyl thiazolyl tetrazolium (MTT) assay, cellular morphology by inverted microscope, the nucleus by Hoechst 33342 staining and cleaved poly ADP-ribose polymerase (PARP) expression by western blot analysis.

**Results:**

Lianzixin extracts (total alkaloids, alcohol extract and water extract) and neferine improved the viability of PC12 cells damaged by caffeine. The morphology of PC12 cells pretreated with neferine, or alcohol or water extract of lianzixin aggregated and attached better than caffeine-damaged cells, but cells pretreated with total alkaloids of lianzixin showed abnormal morphology. Compared with caffeine-damaged cells, cells pretreated with neferine, or alcohol or water extract of lianzixin showed a notable increase in nucleus staining and an obvious decrease in cleaved PARP expression.

**Conclusions:**

Lianzixin extracts and neferine have protective effects against caffeine-induced damage in PC12 cells, which laid a foundation for finding a new medicine value of Lianzixin.

## Background

Caffeine is a well-known xanthine alkaloid and it is one of the most commonly ingested psychoactive substances in the world [1]. It is available in a variety of dietary sources including coffee, tea, cocoa and soft drinks. These are frequently consumed specifically for the stimulant effects of caffeine. Consumption of caffeine is increasing worldwide, with abuse becoming more prevalent [2]. Excessive caffeine ingestion increases the likelihood of adverse nervous effects, such as tachycardia, anxiety and insomnia [3–5]. As a adenosine receptor antagonist, caffeine exhibit therapeutic effect against neurodegenerative diseases [6]. In addition, caffeine induces release of calcium from endoplasmic reticulum, which lead to aberrant calcium homeostasis in neurons [7]. Higher concentrations can promote neuronal neurotoxicity and cell damage and it was widely used in several neurotoxicity models [8, 9]. Given the increasing requirements of daily life and risk of caffeine to human health, the prevention and treatment of caffeine-induced neurotoxicity has become a research target.

*Nelumbo nucifera* Gaertn, a common perennial herb, is widely distributed around the world especially in China, India, Japan and Korea [10, 11]. All parts of *Nelumbo nucifera* Gaertn including the rhizomes, leaves, flowers and seeds can be eaten for their nutritional value and utilized in herbal medicine [12, 13]. Lianzixin is the embryo of the mature *Nelumbo nucifera* Gaertn seed. It is a traditional food and herbal medicine with several nutritional and medicinal value to be found. Principal indications of Lianzixin include treatment of insomnia, nervous disorders, pyrexia and anxiety [14–17]. Lianzixin alkaloids are known to possess a variety of pharmacological activities including antihypertensive, antiarrhythmic and antioxidant effects [18], as well as actions against pulmonary fibrosis, amnesia and cancer [17, 19, 20]. Liensinine, neferine and isoliensinine are the three main alkaloid components of lianzixin that have been shown to have sedative effects in some animal experiments, and neferine is the most abundant alkaloid constituent among them [21]. Since high dose caffeine intake lead to anxiety and insomnia, we speculate that lianzixin or its extracts may possess protective effects against caffeine-induced cellular damage.

The PC12 cell possesses typical features of neuronal cells and is commonly used for in vitro studies on central nervous system diseases [22–24]. Thus, in this work, we used lianzixin extracts (total alkaloids of lianzixin, and alcohol and water extracts of lianzixin) and neferine and undifferentiated caffeine-damaged PC12 cells to identify the effects of Lianzixin on caffeine-induced cellular injury.

## Methods

### Cell culture

PC12 cell is a phaeochromocytoma cell line obtained from rat adrenal glands, it was obtained from Cell Bank of the Chinese Academy of Sciences (Shanghai, China) in the present study and the passage number of PC12 cell was 10. The cells were cultured at 37 °C in a humidified atmosphere containing 5% CO_2_ (Sanyo, Japan). Dulbecco’s modified eagle’s medium (DMEM) (Gibco, USA) supplemented with 10% heat-inactivated fetal calf serum (FCS), 100 IU/mL penicillin and 100 μg/mL streptomycin was the culture medium. Cell monolayers were plated in 6- or 96-well plates (Costar). Cells were digested with 0.25% trypsin (Sigma-Aldrich, USA) and passaged when they reached 70–80% confluence.

### Extraction and isolation

Preparation of lianzixin water extracts: The embryos of lianzixin were purchased from Xiangtan district (Hunan, China). 200 g lianzixin was extracted with water in eight times the volume of the lianzixin for 3 times. Each extraction time was 2 h. The resultant extracts were combined and dried with hypobaric drying method to obtain 60 g of lianzixin water extract, and the percentage yield was 30%.

Preparation of lianzixin alcohol extracts: 200 g lianzixin was extracted by heat-reflux with 80% alcohol in eight times the volume of the lianzixin for 3 times. Each extraction was 2 h duration. The resultant extracts were combined and dried with hypobaric drying method, then 24 g lianzixin alcohol extract was obtained, and the percentage yield was 12%.

Preparation of lianzixin total alkaloids: lianzixin alcohol extracts were diluted with water and then purified using ZTC1 + 1-II clarifying agent (Tianjinzhentiancheng Technology Co., Ltd., China) before elution with distilled water (5 times column volume), 70% alcohol (10 times column volume) and 70% alcohol containing 0.02 g/mL NaCl (7 times column volume) in a cation exchange resin column. Each elution part was collected separately. The product that eluted from the alcohol with NaCl was concentrated, dried and desalted to produce 0.3 g lianzixin total alkaloids, and the percentage yield was 1.88%.

**Fig. 1:**
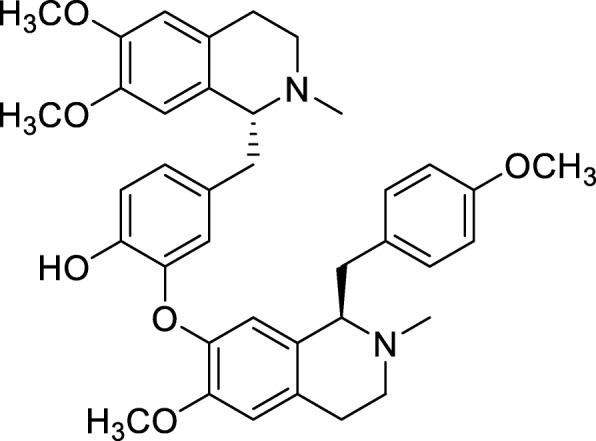
Chemical structure of neferine

**Fig. 2:**
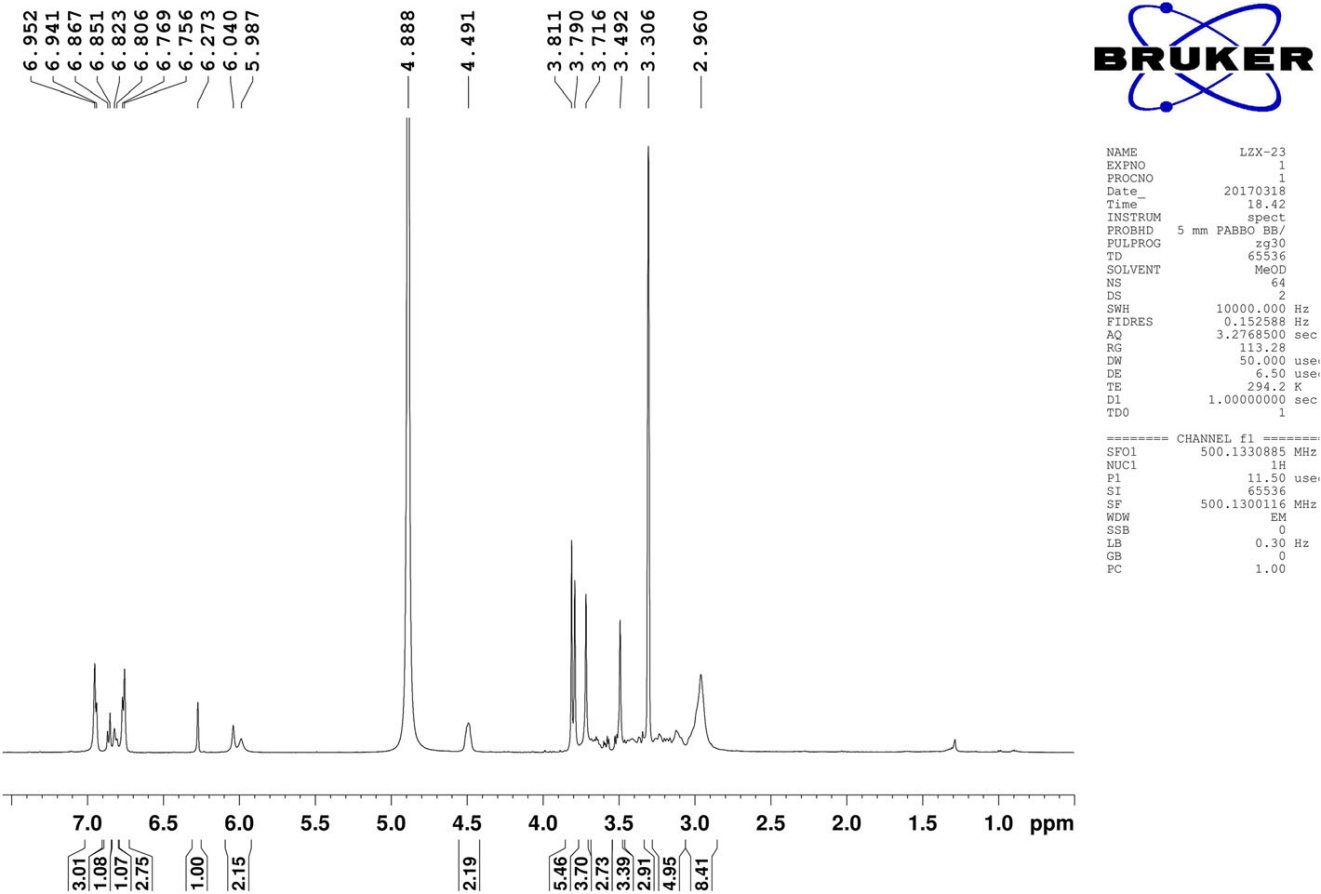
^1^H nuclear magnetic resonance of neferine

**Fig. 3:**
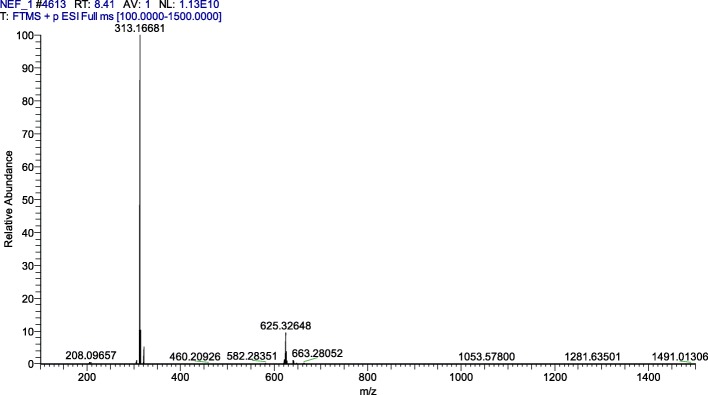
Mass spectrum of neferine

Preparation of neferine: lianzixin total alkaloids were separated using a normal silica gel column gradient elution method. The mobile phase comprised dichloromethane and methanol, which was saturated with triethylamine. There were 26 parts obtained with the ninth part recrystallized with dichloromethane and methanol (1:1) to obtain 20 mg neferine (Fig. 1), and the percentage yield was 0.75%. Neferine was verified by ^1^H nuclear magnetic resonance (^1^H-NMR) (Fig. 2) and mass spectrum (MS) (Fig. 3) [25].

### Experimental design

In the normal control group, cells were cultured in DMEM medium with only dimethyl sulphoxide (DMSO) added. In the caffeine treatment group, a toxic concentration of 5 mmol/L caffeine (Shenzhen phystandard Biotech Co., Ltd., China) was added to the medium [26]. In the lianzixin pretreatment groups, 5 mmol/L caffeine was added after the cells were pretreated with extracts of lianzixin provided by our research group as neferine, total alkaloids of lianzixin, alcohol extract of lianzixin and water extract of lianzixin for 6 h. Dose selection was based on a previous study [27].

### Cell viability assay

MTT (Sigma-Aldrich, USA) assay was used to assess PC12 cell viability and proliferation. In brief, PC12 monolayers were cultured for 24 h after inoculation at a density of 1 × 10^4^ mL^− 1^ in 96-well plates. The cells were then incubated for a further 24 h with different concentrations of the various lianzixin extracts before addition of MTT 5 mg/mL (20 μL) and incubation for 4 h. DMSO 200 μL was next added and oscillated for 20 min. After dissolution of the blue granules, the light absorbance at a wavelength of 490 nm was measured using a semi-automatic microplate reader (BIO-RAD) [28]. Cell viability was expressed as the detected absorbance.

### Cell morphology observation

PC12 cells were inoculated at a density of 6 × 10^5^ mL^− 1^ in 6-well plates and 2 mL culture medium was added to each well. After 24 h, cells were divided into groups according to the different treatments. We chose the protective concentrations of the lianzixin extracts based on the MTT assay results. After treatment with the various compounds for 24 h, the cells were observed and photographed by inverted microscope (Olympus, Japan).

### Hoechst 33342 staining

After observation of morphology, cells were washed with phosphate buffered saline (PBS) and then Hoechst 33342 (0.5 μg/mL) (Sigma-Aldrich) diluted with PBS was added before incubation in the dark at room temperature for 20–30 min. After washing with PBS three times, nucleus dyeing was observed via fluorescence microscope (Olympus, Japan).

### Western blotting analysis of cleaved PARP in PC12 cells

After treatment for 24 h (neferine 1.88 and 6.25 mg/mL, alcohol extracts 3 mg/mL and water extracts 3 mg/mL), cells in the 6-well plates were centrifuged for 3 min at 950 rpm. Precipitates were then collected, washed with PBS and centrifuged again before discarding the PBS. Subsequently, 30–70 μL RIPA buffer supplemented with protease inhibitors were added and maintained on ice for 5 min. Cellular proteins were then scraped with a cell scraper and the protein lysate transferred to 1.5 mL centrifuge tube and maintained on ice for further 30 min. After centrifugation at 13,200 rpm for 15 min, the proteins were collected and the concentrations determined using the Bicinchoninic acid (BCA) method.

The proteins (40 μg) were separated on 10% SDS-PAGE gels. After electrophoresis, they were then transferred onto polyvinylidene fluoride (PVDF) membranes and blocked with 5% nonfat dry milk in Tris-buffered saline. Membranes were incubated with the primary antibodies (MAB374, Cell Signaling Technology, China) overnight at 4 °C. Following this, membranes were washed with Tris-buffered saline containing 0.05% Tween-20 and incubated with appropriate horse radish peroxidase-conjugated secondary antibodies (A9037, Sigma) for 1–2 h at room temperature. The film signal was digitally scanned and then quantified using Image J software.

### Statistical analysis

All data were expressed as the means ± standard error of the means (SEM) and analyzed using SPSS software. Student’s t-test was used to assess the data. *P* < 0.05 was considered statistically significant.

## Results

### The effect of lianzixin on cell viability

**Fig. 4:**
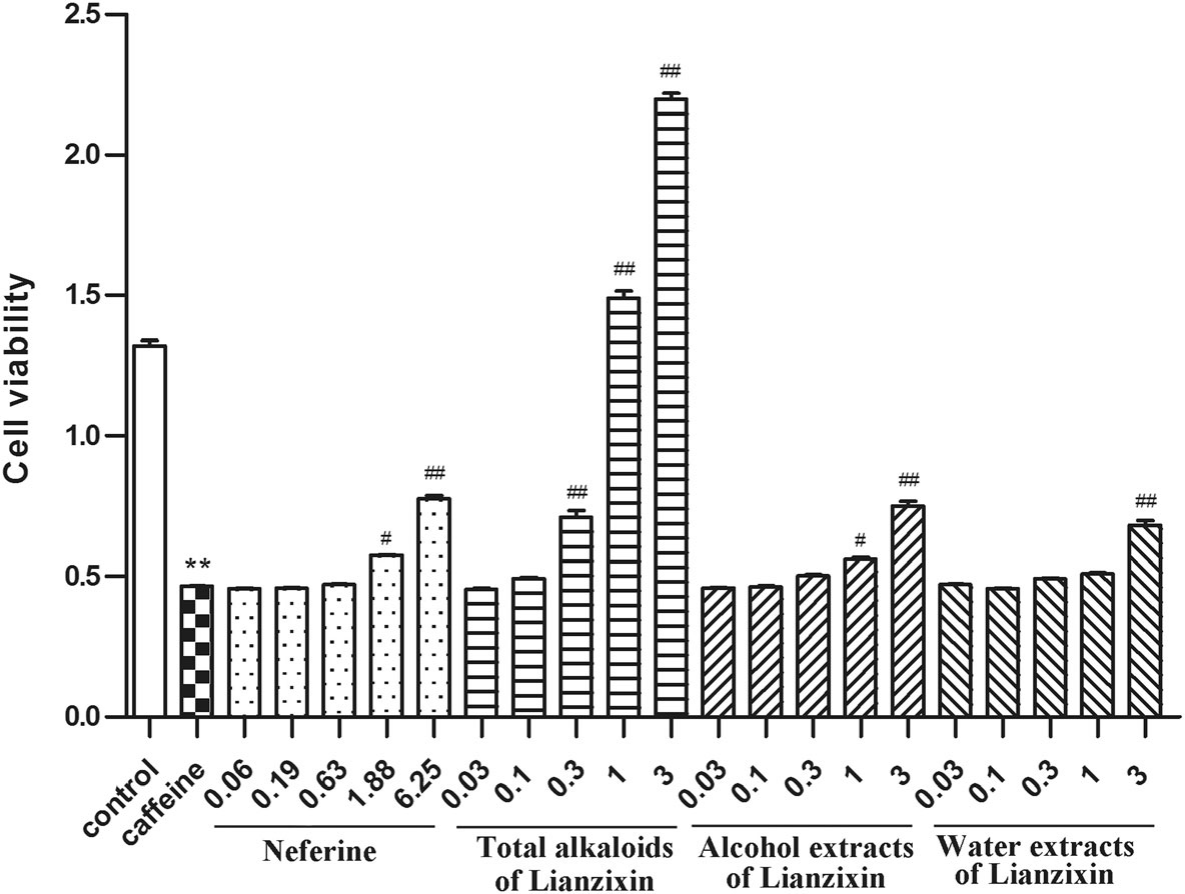
Protective effect of lianzixin extracts on the viability of caffeine treated PC12 cells. Caffeine significantly decreased cell viability compared to the normal control group. Neferine 1.88 and 6.25 mg/mL, total alkaloids 1 and 3 mg/mL, alcohol extract 3 mg/mL, and water extract 3 mg/mL inhibited the decrease in cell viability induced by caffeine. **P < 0.01 caffeine versus normal control group; # *P* < 0.05 lianzixin extracts or neferine versus caffeine treatment group; ## P < 0.01 lianzixin extracts or neferine versus caffeine treatment group

As shown in Fig. 4, caffeine 5 mmol/L significantly decreased PC12 cell viability compared to the normal control group (*P* < 0.01). However, pretreatment with various extracts of lianzixin such as neferine (1.88 mg/mL, P < 0.05 and 6.25 mg/mL, P < 0.01), total alkaloids of lianzixin (1 mg/mL and 3 mg/mL, P < 0.01), alcohol extracts (3 mg/mL, P < 0.01) and water extracts (3 mg/mL, P < 0.01) dose-dependently inhibited the decrease in cell viability induced by caffeine.

### Effect of lianzixin on cell morphology

**Fig. 5:**
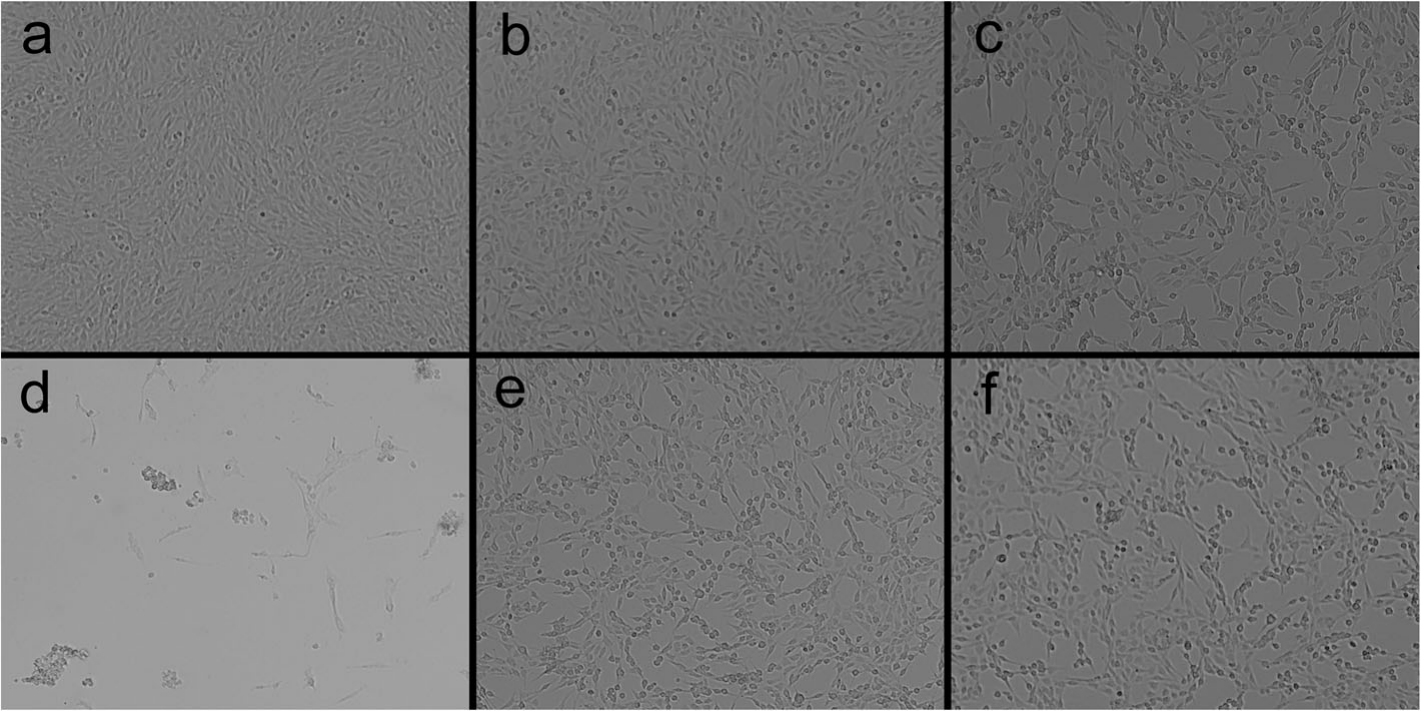
Protective effect of lianzixin extracts on the change in PC12 morphology induced by caffeine. **a** normal control group **b** caffeine treatment group **c** neferine (6.25 mg/mL) treatment group **d** total alkaloids (3 mg/mL) treatment group **e** alcohol extracts (3 mg/mL) treatment group **f** water extracts (3 mg/mL) treatment group

Based on the MTT assay results we chose protective concentrations of the lianzixin extracts as neferine (6.25 mg/mL), total alkaloids (3 mg/mL), and alcohol and water extracts of lianzixin (each 3 mg/mL) to perform the morphology analysis. After treatment with the various compounds for 24 h, the cells were observed and photographed by inverted microscope. As shown in Fig. 5, PC12 cells in the normal control group were in an adhesive situation with a uniform morphology (Fig. 5a). In contrast, caffeine-treated cells had apoptosis and more floating cells (Fig. 5b). Pretreatment with neferine, or alcohol or water extracts of lianzixin restored the morphology of PC12 cells (Fig. 5c, e, and f). In contrast, pretreatment with total alkaloids of lianzixin (3 mg/mL) had detrimental effects on the morphology of PC12 cells (Fig. 5d). Therefore, we elected to abandon further study of the total alkaloids.

### Hoechst 33342 staining

**Fig. 6:**
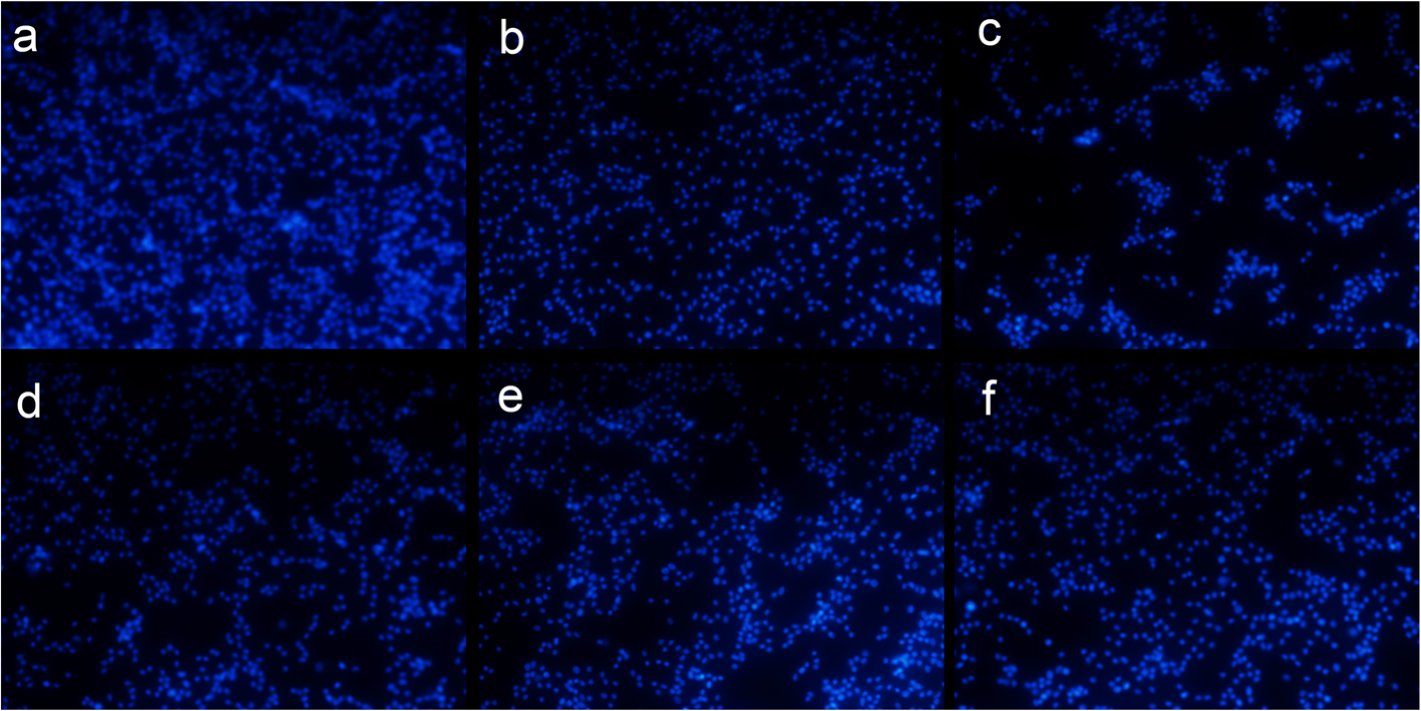
Protective effect of lianzixin extracts on PC12 cells in Hoechst 33342 staining assay. **a** normal control group **b** caffeine treatment **c** Low-dose neferine (1.88 mg/mL) treatment group **d** high dose neferine (6.25 mg/mL) treatment group **e** alcohol extracts (3 mg/mL) treatment group **f** water extracts (3 mg/mL) treatment group

The quantity of cells decreased significantly with caffeine (Fig. 6b) compared to the control group (Fig. 6a). This was not reversed by low-dose neferine (1.88 mg/mL) (Fig. 6c). However, cell numbers in the groups pretreated high dose neferine (6.25 mg/mL) and with alcohol or water extracts of lianzixin (3 mg/mL each) were markedly higher than in the caffeine treatment group (Fig. 6d, e and f).

### The expression of cleaved PARP in PC12 cells

**Fig. 7:**
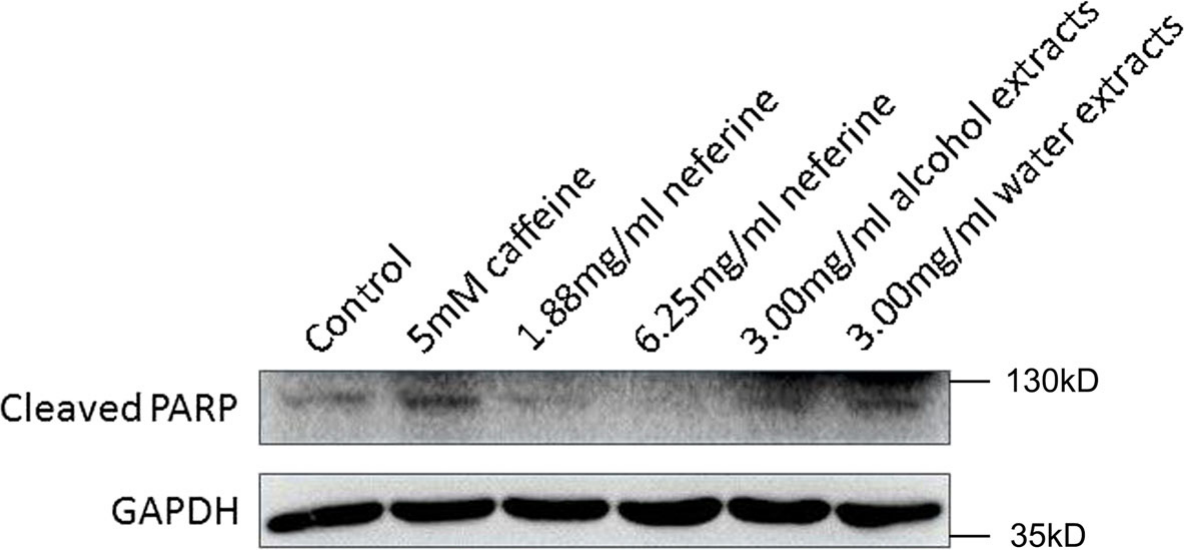
Effect of lianzixin extracts on expression of cleaved PARP in PC12 cells. Caffeine significantly increased the expression of cleaved PARP in PC12 cells. All compounds decreased the expression of cleaved PARP, as compared to caffeine treatment group

**Fig. 8:**
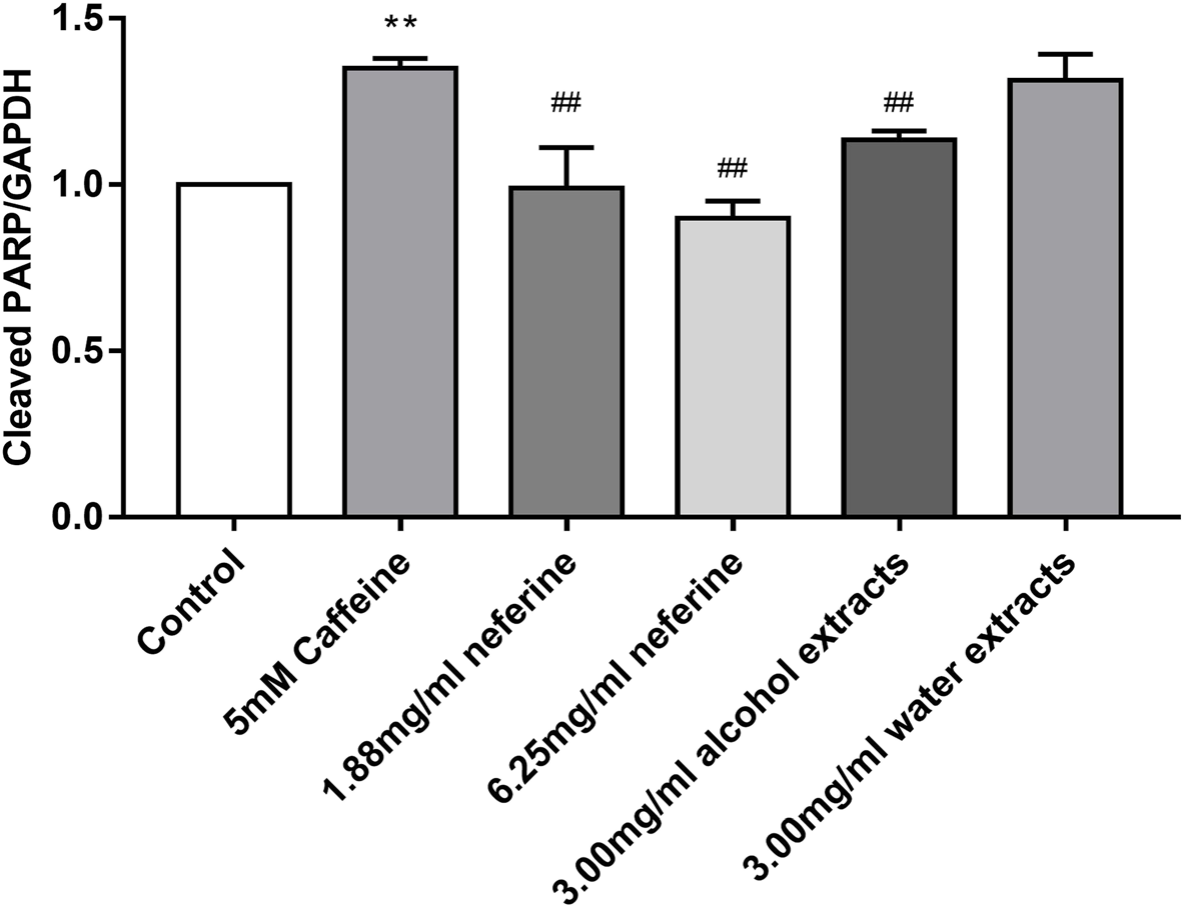
Quantitative data of the expression of cleaved PARP in PC12 cells. **P < 0.01 caffeine versus normal control group; ## *P* < 0.01 lianzixin extracts or neferine versus caffeine treatment group

Treatment with 5 mmol/L caffeine for 24 h significantly increased the expression of cleaved PARP in cells. However, pretreatment with extracts of lianzixin such as neferine (1.88 and 6.25 mg/mL), alcohol extracts (3 mg/mL) and water extracts (3 mg/mL) of lianzixin significantly decreased expression of cleaved PARP induced by caffeine (Fig. 7 and Fig. 8). Furthermore, neferine exerted a more pronounced effect than that of the alcohol or water extracts.

## Discussion

Caffeine is a well-known psychostimulant with several pharmacological activities potentially impacting on basic processes such as sleep, arousal, cognition, learning and memory. Excessive caffeine ingestion increases the likelihood of adverse nervous effects, such as tachycardia, anxiety and insomnia [3–5]. High concentrations of caffeine induce neurotoxicity and cell apoptosis in several cell lines such as human osteoblasts, SH-SY5Y neuroblastoma cell line, cell culture from auditory cortex of rats and cerebellar granular cell culture of rat pups [8, 9, 29, 30]. As a result of these effects, it was widely used in several cytotoxicity models. The toxic concentrations in the above-mentioned cell line vary from about 1 mmol/L to 10 mmol/L. Lianzixin is widely used in Traditional Chinese Medicine for the treatment of high fever with associated restlessness, insomnia, nervous disorders and anxiety. PC12 cell contains many membrane-bound and cytosolic molecules associated with neurons [24] and are widely used for in vitro studies on central nervous system diseases [23, 24, 31, 32]. Events proved that high concentrations of caffeine destructed intracellular calcium homeostasis of PC12 cells, which will lead to cell death [7]. However, to the best of our knowledge, few researches in English have used PC12 damage model induced by caffeine, except a study in Chinese on effect of tanshinone on caffeine-induced PC12 cell injury that proved 10 mmol/L caffeine induced obvious cell injury [33]. Thus, to validate the speculation that neferine and lianzixin extracts may have protective effect on caffeine-induced cellular damage, as well as to ensure the toxic effects of caffeine on PC12 cells, the toxic caffeine concentration of 5 mmol/L and the PC12 cells were applied. Expectedly, results showed that 5 mmol/L caffeine induced obvious cell damage in PC12 cells, which laid a foundation for the use of the cell damage model established by PC12 and caffeine.

There are three major bisbenzylisoquinoline alkaloid constituents in the total alkaloids of lianzixin: liensinine, isoliensinine and neferine, all of which have similar chemical structure [34]. Animal studies showed that all three compounds decreased locomotor activity in mice and possessed sedative effects [21]. However, an unexpected finding in our study was that the total alkaloids of lianzixin did not confer protection for PC12 cells in the cell morphology, possible explanation includes the concentration of total alkaloid used in our study was not proper. At present, studies on the total alkaloids of lianzixin remains insufficient. To the best of our knowledge, there was only one in vitro study that have focused on the protective effect of the total alkaloids on injured cells [35], but few studies have assessed the effective or the toxic concentration for PC12 cells. So further study on different concentration levels is needed. Interestingly, neferine did show apparent protective effect on PC12 cells in the MTT assay and in the Hoechst 33342 staining assay as it exerted greater apparent protective effects than alcohol and water extracts. These findings were consistent with earlier animal work on neferine, where neferine was shown to have significant sedative effect in mice [36]. In addition, the alkaloid constituents of lianzixin were shown in previous research to exert cytoprotective effects against oxidative stress in tert-butyl hydroperoxide injured cells [35]. Besides, it was also demonstrated to attenuates toxicity of mutant huntingtin in PC12 cells [37], which is similar to the observations in our present study. We are the first to demonstrate that neferine and lianzixin extracts have protective effects on caffeine-damaged PC12 cells. This was apparent as increased cell viability, decreased apoptosis, increased cell numbers, and reduced cleaved PARP expression. The results made a foundation for a further study on the protective effect of lianzixin on caffeine-induced neurotoxicity.

Earlier studies have shown that cytosolic calcium homeostasis plays a critical role in keeping normal physiological conditions within nerve cells [38]. It is a complex process affected by factors such as endoplasmic reticulum calcium release and extracellular calcium influx [39]. As an important organelle in eukaryotic cells, the endoplasmic reticulum is a primary source of intracellular calcium and contributes to its regulation [40]. The ryanodine receptor is one of the most important calcium release channels of the neuronal endoplasmic reticulum and it is sensitive to caffeine [41]. After treatment with caffeine, calcium releases into cytoplasm with excessive release leading to an imbalance in calcium homeostasis with subsequent nerve cell injury and apoptosis [42]. For lianzixin extracts and neferine, several studies have described the influence on the Ca2+ transport of cells. In one review article, authors summarized that the antiarrhythmic effect of neferine was mainly related to inhibition of Ca2+ [43]. Another 2007 study on the relaxation mechanisms of neferine on the rabbit corpus cavernosum tissue showed that neferine inhibit extracellular Ca2+ influx and inhibit release of intracellular stored Ca2+ [44]. In our study, it was obvious that caffeine exerted an apparent toxic effect on PC12 cells, and that neferine, alcohol extracts and water extracts conferred protection against this. Therefore, we speculated that the underlying mechanism for the neuroprotective effect may relate to regulation of calcium homeostasis. Further investigations will be required. Aside from neferine, we have not yet determined with accuracy the ingredients in lianzixin extracts that contribute to the protective effect, and the underlying mechanisms for the effect remain to be investigated.

## Conclusions

In summary, we demonstrated that caffeine induced injury in PC12 cells, neferine and extracts of lianzixin including alcohol extracts and water extracts have protective effects on caffeine-injured PC12 cells.

## Data Availability

The data and materials supporting this study are available with the corresponding author upon request.

## Abbreviations

^1^H-NMR: ^1^H nuclear magnetic resonance
BCA: Bicinchoninic acid
DMEM: Dulbecco’s modified eagle’s medium
DMSO: Dimethyl sulphoxide
FCS: Fetal calf serum
MS: Mass spectrum
MTT: Methyl thiazolyl tetrazolium
PARP: Poly (ADP-ribose) polymerase
PBS: Phosphate buffered saline
PC12: Phaeochromocytoma cells
PVDF: Polyvinylidene fluoride
SEM: Standard error of the means
